# Excision of the Cervix Uteri for Corroding Ulcer

**Published:** 1856-03

**Authors:** Laurence Turnbull

**Affiliations:** Philadelphia


### *Excision of the Cervix Uteri for Corroding Ulcer.—Recovery.*

By LAURENCE TURNBULL, M. D., Philadelphia.

Martha H., aged twenty-five years, unmarried, has never enjoyed good health, and has always been irregular at her menstrual periods. She had a severe attack of dysentery in 1852, and upon recovery suffered with constant pain in the lower part of the abdomen and region of the uterus, with a sensation of weight and leucorrhœal discharge. She placed herself under the care of a physician who treated her for prolapsus uteri, by means of rest, injections, &c., and an abdominal supporter. She remained under his charge for one month without benefit, the supporter increasing her pain so much that she was unable to wear it. Obtaining no relief, she applied to a second physician, who examined her by touch, and ordered strong injections of nitrate of silver, with some internal remedies, which she employed, but with no amelioration of the pain from which she suffered.

In May, 1853, she came under my care; at that time her menses were slight, and irregular, with occasional vicarious discharges of blood from the stomach; her pain was chiefly in the region of the womb, passing down the limbs; skin pale and anemic, appetite poor, and bowels constipated. I prescribed iron as a tonic, with a gentle aperient, and narcotics to relieve the pain, but made no examination, she not being willing. She continued the use of these medicines for several months, her general health becoming somewhat improved, but still her pain continued.

Towards the end of 1853 she suffered so much, and was so harassed by it, that she reluctantly consented to a more careful examination by the touch and speculum, which brought to view a gray colored ulcer penetrating the neck of the os uteri, and causing it to turn towards the rectum, the neck being also increased in volume.

The treatment pursued was rest, cupping over the sacrum, leeches to the lower part of the abdomen with an occasional blister, dressed with mercurial ointment, and the internal use of small doses of calomel, with solid nitrate of silver applied to the ulcer; mucilaginous baths and anodyne injections were also used.

By these means she was made much more comfortable, but the ulcer would not heal; still she was able to engage in her usual occupation.

In January, 1855, I was again called to visit her in haste, on account of an excessive hemorrhage which had taken place from her stomach. This discharge was of more frequent occurrence, also, than before, her menstrual discharge having almost entirely ceased. Fearing the loss of so much blood I prescribed small doses of acetate of lead and opium, which seemed to moderate the flow of blood and diminish her pain.

I now gave her small doses of bichloride of mercury until it produced salivation, and touched the ulcer with the proto-nitrate of mercury, which, however, produced no permanent benefit. After the effects of the mercury had passed away, I placed her upon the internal use of large doses of extract of conium in combination with the iodide of potassium, and changed the local application to the ulcer, but with little advantage to my patient, as she was relieved only for the time, the pain returning with such violence all down her limbs and side as to render her unable to walk, so that she was obliged to give up her work.

She now desired to know if it were not possible to do something more for her, for she remarked, "she was not able to bear the pain which she suffered." I told her the only means left was the removal of the neck of the womb by the knife; she said she was perfectly willing.

Having friends and relations, I gave her time for consideration and consultation, and fully explained the uncertain results and difficulties of the operation; she still urged that her life was a burthen to her, and she was willing to run the risk of the operation for the chance of a cure.

On May 21, 1855, I proceeded to operate in the following manner, assisted by my friend Dr. Hewson Bache. Placing the patient on the edge of a bed resting on her knees in the position recommended by Dr. Sims for vesico-vaginal fistula, ether was administered to her, and a metal speculum was placed in the rectal portion of the vagina with one on each side, so as to expose fully the neck of the womb.

Having cleansed the parts by means of a pallet of cotton, the double pincers of Museux were carried close up to the os tincæ, which I seized from before backwards as high as possible; I then commenced a slow and sustained traction until the os was brought to the exterior orifice; (being under the influence of the ether, this gave her little pain); then with the curved knife of Jobert cutting on its concave edge, and holding the pincers with my left hand, I cut through the healthy tissue slowly from left to right, until all the ulcerated and enlarged portions were removed, making a concave even cut of the neck of the womb.

The hemorrhage was very abundant, and poured out of the vagina in a stream for some time; this was checked by plugging the vagina with pieces of old muslin, which were secured by a T bandage. She was then placed upon her side and directed to take one grain of opium every two hours, with iced gum water as drink.

In the afternoon she suffered from severe nervous spasms, which were relieved by the use of sulphuric ether and opium.

May 22.—Was called at four o'clock, A. M., to relieve the bladder and remove the tampon; had taken twelve grains of opium through the night, was restless and still suffered pain. Skin dry, pulse 84, syringed the parts with flaxseed tea, and directed opium to be continued with oat meal gruel as diet.

May 23.—Pain less, slept without the opium, pulse 83, tongue furred. Ordered pil hydr. gr. iij., pulv. opii. gr. j. ℞ ft. pilula No. 1. To be taken at night, and followed by ol. ricini in the morning. She continued to suffer a good deal of pain, and required daily attendance until July, when she had a severe attack of convulsions which lasted nearly twelve hours. Towards the end of July she was able to be removed to the country, where she gradually improved, and returned in August looking and feeling well, with a good color and appetite, and quite able to resume her usual occupation in a cotton mill.

The womb looked healthy, the parts had healed, and menstruation had returned, with no hemorrhage from the stomach.

In the above case every means were employed to relieve previous to the operation and all had failed.

She had taken in large doses, opium, morphia, ether, chloroform, and many of the antispasmodics; mercury, arsenic and iodine had also been administered internally, so as to produce their specific effects, but without benefit; various local applications had been tried in vain, such as the salts of lime, lead, silver, copper, solution of soot, nitric acid and nitrate of mercury, and the actual cautery when every other application failed in relieving her.

The pain was that of cancer, being "gnawing" and so intense that she was perfectly willing to take any thing or do any thing to be relieved, and therefore I considered myself perfectly justified in trying the effect of an operation before the whole system became involved.

January 25, 1856.—Martha called to see me; she still suffers some pain at certain periods, but has gained several pounds in weight, and is able to live with much more comfort than before the operation.

In the microscopic examination of the specimen removed by Dr. Da Costa of this city, nothing was found but the normal cells hypertrophied, and fibro-plastic cells.

*February*, 1856.

